# Chemical Diversity as a Function of Temperature in Six Northern Diatom Species

**DOI:** 10.3390/md11114232

**Published:** 2013-10-30

**Authors:** Siv Huseby, Maria Degerlund, Gunilla K. Eriksen, Richard A. Ingebrigtsen, Hans Chr. Eilertsen, Espen Hansen

**Affiliations:** 1 MabCent-SFI, University of Tromsø, N-9037 Tromsø, Norway; E-Mail: siv.huseby@uit.no; 2 Department of Arctic and Marine Biology, Faculty of Biosciences, Fisheries and Economics, University of Tromsø, N-9037 Tromsø, Norway; E-Mails: maria.degerlund@uit.no (M.D.); gunilla.eriksen@uit.no (G.K.E.); richard.a.ingebrigtsen@uit.no (R.A.I.); hans.c.eilertsen@uit.no (H.C.E.); 3 Marbio, University of Tromsø, N-9037 Tromsø, Norway

**Keywords:** diatoms, physiology, metabolic fingerprinting, temperature

## Abstract

In this study, we investigate how metabolic fingerprints are related to temperature. Six common northern temperate diatoms (*Attheya longicornis*, *Chaetoceros socialis*, *Chaetoceros furcellatus*, *Porosira glacialis*, *Skeletonema marinoi*, and *Thalassiosira gravida*) were cultivated at two different temperatures, 0.5 and 8.5 °C. To exclude metabolic variations due to differences in growth rates, the growth rates were kept similar by performing the experiments under light limited conditions but in exponential growth phase. Growth rates and maximum quantum yield of photosynthesis were measured and interpreted as physiological variables, and metabolic fingerprints were acquired by high-resolution mass spectrometry. The chemical diversity varied substantially between the two temperatures for the tested species, ranging from 31% similarity for *C. furcellatus* and *P. glacialis* to 81% similarity for *A. longicornis*. The chemical diversity was generally highest at the lowest temperature.

## 1. Introduction

The general biochemical composition of algae is relatively well described in terms of common compounds [1,2,3]. The overall elemental composition of phytoplankton usually mirrors the elemental composition in deep ocean seawater. This is termed the “Redfield stoichiometry”; C:N:P = 106:16:1 [4]. These ratios are means, and in practice, they may vary considerably. When the concentrations and ratios of the elements in surface waters vary, the same pattern can often be seen in the phytoplankton [5].

From studies applying metabolomic approaches, it is also known that both environmental and physiological conditions can cause the overall biochemical content to change [6,7,8]. Diatoms are known to have a rapid evolution compared to other groups of organisms [9]. Changes in environmental factors can induce rapid phenotypic adaptions in diatoms, and therefore the emergence of physiologically different strains can take place at relatively short timescales [10,11].

Several studies of the chemical composition of diatoms as a function of temperature have been done, although some of them have led to contradictory conclusions. It is e.g., a common conception that volume specific C and N content varies with temperature, and decreases with increasing cell size [12,13]. This finding is disputed by others who claim that volume specific C and N contents are not affected by temperature [14]. Lipid composition may change with temperature [15], and a number of metabolites may well differ between exponential, stationary and declining growth phases, and there may also be diurnal variations during the exponential phase [16].

Metabolomics is a relatively young discipline, and there are not many examples in the literature where this approach has been used to study diatoms. However, there are some studies that give strong indications of large chemical diversity within same species at varying environmental conditions, between species [17] and also between strains of the same species [18]. The diatom genomes are large, and several species have been genome sequenced, such as the centric diatom, *Thalassiosira pseudonana* (32.4 Mb), and the pennate diatom *Phaeodactylum tricornutum* (27.4 Mb) [6,19]. Large amounts of “functionally unknown” sequences were found, indicating that the diatoms might have potentials for metabolic flexibility and high chemical diversity. This is supported by the fact that diatoms are known to produce a wide array of secondary metabolites [19].

The Arctic is often inhabited by Atlantic warm water species that have adapted to the lower temperatures and act as psychrophiles. They are actually genetically distinct groups of the same morpho-type, as in the case of *Chaetoceros socialis* [20]. It is also common that larger species are present during the late part of the annual spring bloom while smaller species make up the initial stock of the bloom [21]. This means that the different species multiply at highly variable temperatures. In fact March-April temperature anomalies are of the order 4 °C between years, meaning that if the chemical content of diatoms are influenced by temperature, this may have significant ecological effects.

In the present study, we wanted to investigate how the chemical diversity of six diatom species was affected by their growth temperature. We used high-resolution mass spectrometry to obtain metabolic fingerprints of two size classes of monoclonal diatom cultures kept at two different temperatures. The analysis of the data was focused at differences and similarities in mass-to-charge (*m/z*) signals between samples rather than on the specific identity of compounds.

## 2. Results and Discussion

Monocultures of six common northern temperate centric diatom spring bloom species (Table 1) were cultured at two different temperatures, 0.5 and 8.5 °C.

**Table 1:** Species and applied acronyms (in brackets), strain IDs, duration of cultivation period for the two temperatures 0.5 and 8.5 °C and geographical origin (latitude °N).

Species	Strain ID	Duration (days) 0.5/8.5 °C	Origin
*Attheya longicornis* (Al) Crawford and Gardner	AMB20.1	14/10	North Norwegian coast (69.5)
*Chaetoceros socialis* (Cs) Lauder	AMB80	21/10	Barents Sea (74.5)
*Chaetoceros furcellatus* (Cf) Bailey	AMB61	10/10	Barents Sea (77.8)
*Skeletonema marinoi* (Sm) Sarno and Zingone	AMB39	17/10	North Norwegian coast (69.5)
*Thalassiosira gravida* (Tg) Cleve	AMB85	10/10	Ramfjord (69.4)
*Porosira glacialis* (Pg) (Grunow) Jørgensen	AMB49.2D	10/10	Tromsøysund (69.4)

The six species investigated in our study all belongs to the centric diatoms. This group has been revealed not to be monophyletic (species with shared characteristics) and thus do not reflect a natural classification. It has been shown that morphological symmetry is an ambiguous character for classification and hence biochemical functioning. In a phylogenetic tree based on SSU rDNA sequences the six species we have used are grouped as follows: the genera *Thalassiosira*, *Skeletonema* and *Porosira* are in one group, the *Chaetoceros* in another while *Attheya* is a separate group close to the pennates. These groupings are in general also supported by other studies [21,22]. In terms of cell size the species belongs to two classes, *i.e.*, *A. longicornis*, *C. socialis*, *C. furcellatus* and *S. marinoi* are small species (cell volumes < 300 µm^−3^) while *P. glacialis* and *T. gravida* are large species (cell volumes 8000–35,000 µm^−3^).

### 2.1. Growth Rates

When the experiments started, cell concentrations were 150,000–350,000 cells L^−1^ and the Chl*a* concentrations varied between 0.2 and 0.7 µg L^−1^ for the small cells and between 5.0 and 9.6 µg L^−1^ for the large cells. Growth rates during the exponential phase (calculated from the two last samplings) ranged between 0.29 and 0.48 doublings day^−1^ for cells cultivated at 0.5 °C (Table 2). The mean value for this temperature was 0.36 doublings day^−1^. The largest cells had slightly lower growth rates than the small ones. The cells cultivated at 8.5 °C had mean growth rates of 0.52 doublings day^−1^, and it was the largest species, *i.e.*, *P. glacialis*, that had the lowest growth rate.

**Figure 1:**
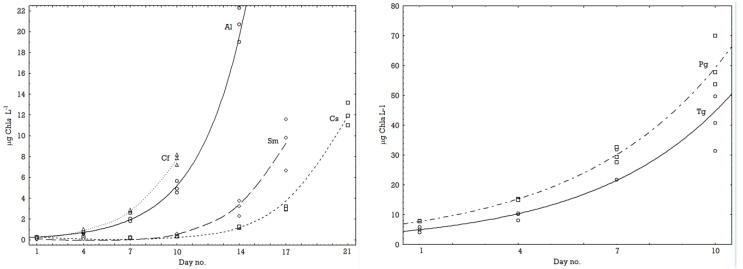
Rate of increase in biomass at 0.5 °C of the small species (left) *A. longicornis* (Al), *C. socialis* (Cs), *S. marinoi* (Sm) and *C. furcellatus* (Cf), and the large species (right) *T. gravida* (Tg) and *P. glacialis* (Pg). The lines drawn are exponential curves fitted to the three replicate measurements from each sampling.

**Figure 2:**
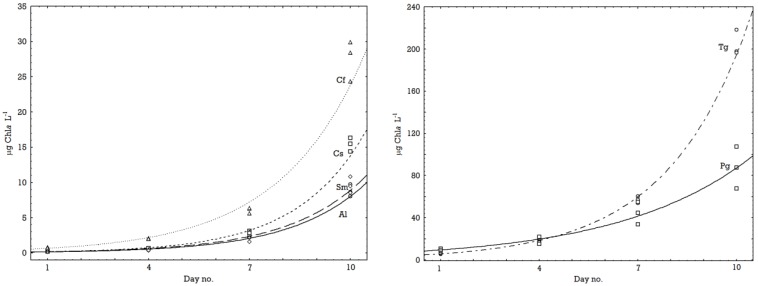
Rate of increase in biomass at 8.5 °C of the small species (left) *A. longicornis* (Al), *C. socialis* (Cs), *S. marinoi* (Sm) and *C. furcellatus* (Cf) and the large species (right) *T. gravida* (Tg) and *P. glacialis* (Pg). The lines drawn are exponential curves fitted to the three replicate measurements from each sampling.

At 0.5 °C it was *C. furcellatus* followed by *A. longicornis* that had the fastest growth, while at 8.5 °C the fastest growers were *C. socialis* and *S. marinoi*. As judged from the growth curves (Figure 1 and Figure 2) all species at all temperatures were in an exponential growth phase at the end of the experiments. The *Fv/Fm* values increased up till day 4 to 7 of the experiment and then became stable (Table 2), *i.e*., the values were always higher at the end of the experiment than at the start (Table 2). Samples for metabolomics (MS) analysis were taken at the last day of the experiments when the cultures were in an exponential growth phase (Figure 1 and Figure 2).

**Table 2:** Growth rates (doublings day^−1^) and *Fv/Fm* calculated as mean of three replicates at start and end of the experimental period for the six investigated diatoms *vs.* temperature.

°C	Species (Acronyms)	µ-Chl*a*	*Fv/Fm* start	*Fv/Fm* stop
0.5	*Attheya longicornis* (Al)	0.44	0.598	0.674
0.5	*Chaetoceros socialis* (Cs)	0.27	0.452	0.683
0.5	*Chaetoceros furcellatus* (Cf)	0.48	0.631	0.720
0.5	*Skeletonema marinoi* (Sm)	0.39	0.489	0.694
0.5	*Thalassiosira gravida* (Tg)	0.30	0.645	0.711
0.5	*Porosira glacialis* (Pg)	0.29	0.714	0.734
0.5	Mean for temperature	0.36	0.588	0.703
8.5	*Attheya longicornis* (Al)	0.56	0.736	0.674
8.5	*Chaetoceros socialis* (Cs)	0.62	0.627	0.738
8.5	*Chaetoceros furcellatus* (Cf)	0.52	0.704	0.736
8.5	*Skeletonema marinoi* (Sm)	0.57	0.445	0.692
8.5	*Thalassiosira gravida* (Tg)	0.51	0.670	0.715
8.5	*Porosira glacialis* (Pg)	0.32	0.711	0.711
8.5	Mean for temperature	0.52	0.649	0.711

Gilstad and Sakshaug [23] monitored the maximum growth rates of 10 temperate diatoms at 0.5 °C. They achieved a mean doubling rate of 0.46 doublings day^−1^, and *C. furcellatus* was amongst the slowest growers of the ten species they checked. In our experiments, *C. furcellatus* was the fastest grower overall, and particularly fast at 0.5 °C. Gilstad and Sakshaug [23] also concluded that species-specific growth strategies could not be detected.

It has been argued that growth rates of diatoms are related to the cell size and that smaller cells have higher growth rates than large ones [5]. This is not clearly supported by our data. *P. glacialis*, the largest species in the study did indeed show low growth rates at both temperatures, but the second largest species; *T. gravida*, divided fast at 8.5 °C (Table 2) and the small species *C. socialis* was relatively slow at 0.5 °C. Growth rate is also reported to be affected by life cycles in diatoms [24], which we cannot rule out as a possible contributing factor also in the present study.

The use of maximum quantum yield as a measure of growth status (nutrient–light limitation) has been debated heavily [25]. The highest values were for *P. glacialis* at both temperatures, while in fact this species was the slowest grower at 8.5 °C, and the second slowest at 0.5 °C. We will not discuss the reasons for this here but just conclude that our logged maximum quantum yield values were well above what is normally considered to be indicative of microalgae in resource-limited growth [25]. Our results showed that the overall growth rates obtained at the two temperatures did not differ as much as expected. Growth rates are expected to increase linearly or exponentially, with increasing temperature [26,27]. The obtained growth rates at the lower temperature are in accordance with the Eppley equation [27] if corrected for the photoperiod regime we used. However, at the high temperature (8.5 °C) at least 0.7 doublings day^−1^ was to be expected [27,28]. One reason why we did not obtain such growth rates in our experiments may be that the species we used were northern clones, and as such were adapted to low optimum temperatures. In order to judge if a species has entered the exponential phase, it is the rate of increase that matters and not the biomass concentration obtained because small cells will reach lower biomass values than large cells after equal number of doublings. Nevertheless, we sampled the diatoms in the exponential phase at comparable growth rates at different temperatures, and this allowed us to examine the metabolic profiles of the species *vs.* temperature [28].

### 2.2. Chemical Composition

The diatom samples were extracted in 70% aqueous methanol prior to analysis by MS. We chose this solvent because it is directly compatible with electrospray ionization (ESI). This allows us to avoid any evaporation or re-suspension steps reducing the risk of degradation of unstable compounds. No extraction protocol will provide the full suite of metabolites present in the diatom samples, however, the extraction using 70% aqueous methanol will give a broad set of metabolites that give good signals in ESI-MS and therefore provides a good basis for comparison of metabolic profiles.

**Figure 3:**
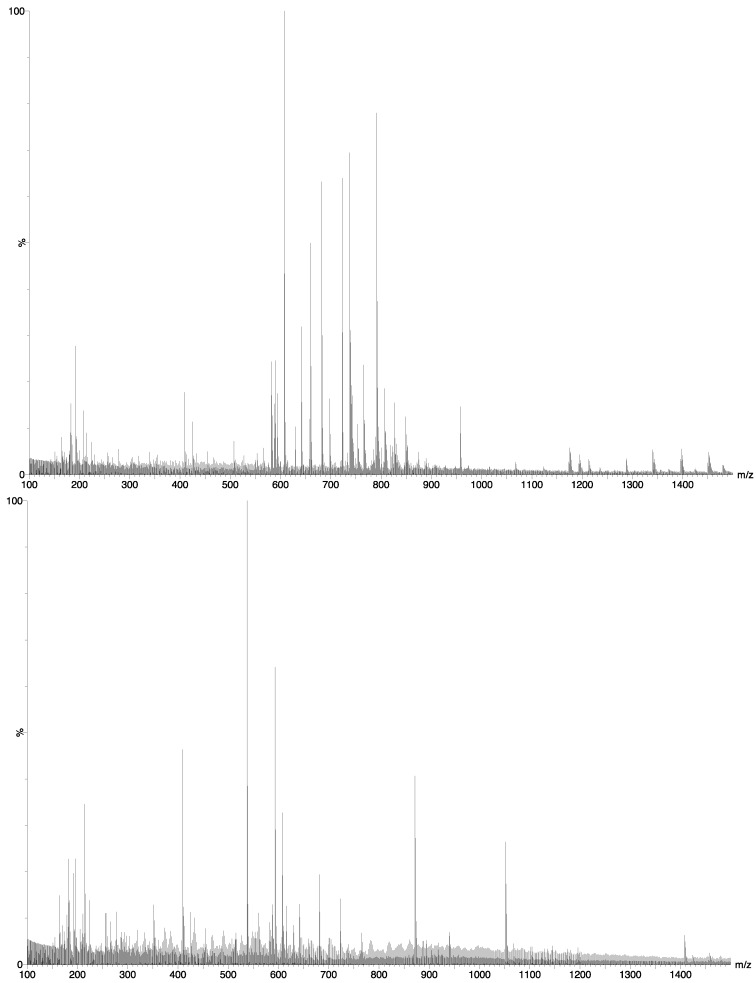
Mass spectra of two of the samples: *P. glacialis* (top) and *S. marinoi* (bottom), both cultivated at 8.5 °C.

Metabolic profiles of the diatom extracts were generated by using flow injection high-resolution electrospray MS (HR-ESI-MS) data (Figure 3). One minute of data from each sample was averaged at two decimals precision level. Each genuine *m/z* value was regarded as a unique metabolic marker.

**Figure 4:**
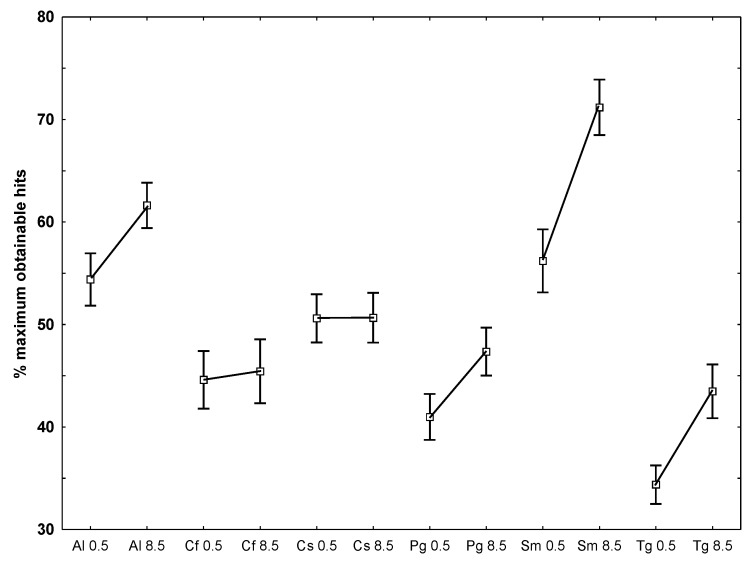
The number of common markers as a % of the maximum obtainable when each replicate from each species and temperature (0.5 and 8.5 °C) was compared to all other replicates (all species). Vertical bars are ±1 SE and *n* = 54 for each species-temperature. Lines drawn between points only serve to delineate the species.

The number of metabolic markers included from each analyzed sample after screening of data (see Experimental Section 3.4) ranged from *ca.* 1000 to 1250. Our data analysis led to the following conclusions:

I. When we tested the number of similar markers within one species (*P. glacialis*) for each temperature separately the mean value was *ca.* 90% (Table 3).
II. When the markers from each temperature (all species included) were tested against each other (independent students *t*-test), the two groups proved statistically different at *p*-level 0.00013 (mean 0.5 °C = 46%, 8.5 °C = 55%).
III. Tests where replicates from one temperature were tested against replicates from the other temperature for each species separately showed a mean similarity in markers of *ca.* 60%. The species that differed most in detected markers were *P. glacialis* and *C. furcellatus* (both 31%) and *C. socialis* (52%). The species that had the highest similarity in markers were *S. marinoi* (92%), *A. longicornis* (81%) and *T. gravida* (74%) (Table 3).
IV. To examine which of the temperatures that caused the largest deviation from the optimal obtainable 90% similarity, the number of hits for each species at each temperature against all other species at both temperatures, it appeared that lower hit rates at the lowest temperature was present for *A. longicornis*, *P. glacialis*, *S. marinoi* and *T. gravida* while the two *Chaetoceros* species did not differ between temperatures (Figure 4).

**Table 3:** Similarity between replicates of MS samples for *P. glacialis* at each temperature, and for replicates from 0.5 °C tested against replicates from 8.5 °C for each species separately.

°C	Species	*n*	% of maximum obtainable hit number (SE in %)
0.5	*Porosira glacialis* (Pg)	36	91.3 (2.1)
8.5	*Porosira glacialis* (Pg)	36	88.4 (2.9)
0.5 *vs.* 8.5	*Attheya longicornis* (Al)	9	81 (2.7)
0.5 *vs.* 8.5	*Chaetoceros socialis* (Sc)	9	41 (3.3)
0.5 *vs.* 8.5	*Chaetoceros furcellatus* (Cf)	9	31 (2.2)
0.5 *vs.* 8.5	*Skeletonema marinoi* (Sm)	9	92 (4.7)
0.5 *vs.* 8.5	*Thalassiosira gravida* (Tg)	9	74 (3.1)
0.5 *vs.* 8.5	*Porosira glacialis* (Pg)	9	31 (6.5)

Our interpretation of the data is that a change in cultivation temperature induces synthesis of new chemical compounds (II), and that these changes may be of significant magnitude (III). For e.g., *P. glacialis* and *C. socialis* 40%–50% of the markers were due to different cultivation temperatures. *S. marinoi* showed no difference between temperatures while *A. longicornis* showed a minor difference (*ca.* 10%). There are also indications that the lowest temperature induced synthesis of more diverse compounds (IV). It is also interesting to note that we could not observe any discernable size effects since, e.g., both the largest species (*P. glacialis*) and the smallest one (*C. socialis*) showed large variations between temperatures.

One of the limitations of the method we used to acquire the MS data (FIE-MS) is that the compounds in the extract are not separated in time before they enter the MS and the isotopic pattern of individual compounds is impossible to recognize. It is therefore not feasible to identify specific compounds from the data available through the present study, and we cannot say what kind of metabolites that varies between the different species and temperatures.

## 3. Experimental Section

### 3.1. Cultivation of Diatom Samples

Monocultures of six common northern centric diatom spring bloom species (Table 1) were used in the present experiment. The cultures originated from single cells or chains of the specimens isolated from multi-species diatom cultures. These cultures had been started by germinating spores contained in bottom sediments or from water samples collected in the Barents Sea and along the coast of northern Norway (Table 1). The species *Attheya longicornis*, *Chaetoceros socialis*, *Porosira glacialis* and *Skeletonema marinoi* were identified by applying morphological and molecular methods (18s rDNA, (SSU) and large subunit, 28s rDNA (LSU)) [20]. The species *Thalassiosira gravida* and *Chaetoceros furcellatus* were morphologically identified from cleaned frustules and spores using light microscopy.

The cultures were kept in well used and thoroughly cleaned/sterilized 1.5 L soft drink (PET) plastic bottles at two temperatures (0.5 °C and 8.5 °C) at the same scalar irradiance (25 µmol m^−2^ s^−1^, PAR), in temperature and light (digitally) controlled rooms. Light was measured with a QSL-100 (Biospherical Instruments Inc., San Diego, CA, USA) sensor. Illumination was fluorescent tubes (Osram L 58W/954 Daylight) and photoperiod was L:D; 14:10. To ensure that the cultures received the same amount of light, the positions of the bottles were randomly altered on a daily basis, and the cultures were carefully mixed. The experiment was set up with three replicates (A, B, C) of each of the six species at both temperatures (Table 1). Prior to the start of the experiment, the cultures were grown under the experimental light regime and the two temperatures for one week. At the initiation of the experiment, the stock cultures were diluted to concentrations between 150,000 and 350,000 cells L^−1^. Cultivation took place in nutrient sufficient medium prepared from filtrated and autoclaved (deep) water from Malangen outside Tromsø (−160 m, 69°29′73″ N, 18°23′60″ E). Guillard’s f/2 Marine Water Enrichment and silicate solution were added to a final concentration equivalent to f/10.

Samples for cell counts were taken at start of the experiment and fixated with 2% Lugol’s acid solution. Cells were enumerated in a Leitz inverted microscope after at least 2 hours settling time in Nunc 4 well 2 mL sedimentation chambers. To obtain a measure of cell biovolume 10 cells from each sample were measured (diameter and length) and the mean sizes were used to calculate the biovolumes [29]. Chl*a in vitro* was measured approximately twice weekly during the experiment period. We used a Turner TD-700 fluorometer applying the method in Holm-Hansen and Riemann [30] using methanol as extraction solvent. For *in vitro* Chl*a* analysis, samples were filtrated and left to extract in dark for at least 24 h. Growth rate was calculated from the Chl*a* values as doublings per day, see Equation (1).



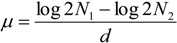
(1)
*N*_1_ and *N*_2_ are Chl*a* at start and end of measurement period, respectively, and *d* is the number of days between *N*_1_ and *N*_2_. As a measure of physiological status photosynthetic efficiency as maximum quantum yield in Photo System II was measured using a Water-Pulse Amplitude Modulated (PAM) fluorometer (Water-ED/B, Heinz Waltz GmbH, Effeltrich, Germany). Maximum quantum yield (mol electrons × (mol photons absorbed)^−1^) was measured twice weekly. At sampling time 2.5 mL from each bottle was transferred to the Water PAM quartz cuvette. Samples were dark adapted for 3 min (5 µs measuring light pulses applied at a frequency of 18 Hz) and then a yield measurement with a saturating light pulse (peaking at 660 nm and a frequency of 20 kHz) was performed. Maximum quantum yield was calculated as:

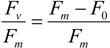
(2)
where *F_m_* is the maximum fluorescence from the saturating light pulse in dark acclimated cells and *F*_0_ is the initial fluorescence (from the measuring light) in dark acclimated cells. Since the different species showed variable grow rates, the cultures were terminated at different dates to ensure they were in the exponential growth phase (Table 1). Material for mass spectrometry analyses was collected onto burnt (540 °C, 8 h) GF/C 47 mm filters until the filters were clearly colored. A sample of the growth medium used was also treated in the same manner (blank sample). The filters were immediately after filtration shock frozen in liquid nitrogen and thereafter stored at −77 °C in a bio freezer until further analysis.

### 3.2. Extraction and Preparation for Mass Spectrometry (MS)

The GF/C-filters containing the cells were transferred to centrifugal tubes and 6 mL 70% aqueous methanol was added. The tubes were sealed with lids, and incubated by shaking in darkness for four hours at 4 °C, thereafter the tubes were centrifuged at 4000 rpm at 5 °C for 15 min. Aliquots of 1 mL from each extract were transferred to a deep-well plate, followed by the addition of 5 µL 10% formic acid before the plate was sealed and stored at −20 °C until analyzed by MS.

### 3.3. Flow-Injection Electrospray Mass Spectrometry (FIE-MS)

Mass spectra were acquired using a Waters LCT Premier (Milford, MA, USA) time-of-flight mass spectrometer equipped with an electrospray ion source and controlled by MassLynx 4.1. The crude extracts were introduced to the mass spectrometer using a Waters 2795 Alliance HT HPLC without any column. The HPLC pumped a mobile phase consisting of 50% aqueous acetonitrile containing 0.05% formic acid at a flow rate of 50 µL min^−1^ into the ion source, and aliquots of 50 µL of the extracts were loaded into the flowing mobile phase. The mass spectrometer was operated in the W-mode (both reflectrons active), and at capillary and cone voltages of 2600 and 70 V, respectively. The desolvation chamber was kept at 250 °C and the ion source at 150 °C, while the desolvation gas flow rate was 300 L h^−1^ and the nebulizer gas flow rate was 5 L h^−1^. Leucine-enkephalin was infused through the reference probe and used as lock mass for internal calibrations throughout the data acquisitions. Every morning when the analysis took place, the instrument was tuned to a resolution of at least 10,000 FWHM and calibrated using sodium formate. Data was acquired for one min in the positive ion (ES+) mode, and the mass range was set to 100–1500 *m/z*. The samples were not run all at the same round. The samples from the A bottles of all species as well as from the B bottles of *P. glacialis* were run the first round, the remaining samples three months later. One injection was performed for each sample.

### 3.4. MS-Data Analysis

In order to compare samples with respect to detected markers (*i.e*., *m/z* signals), without focusing on the actual identity of the molecules, we applied a self-developed software application (programmed in C+). We removed background noise by excluding *m/z* signals with intensities less than 7% of the base peak. Markers found in the blank sample were also excluded from the dataset. The program compared all markers in each sample (direct numerical comparison) with all markers in the other samples and arranged this in a data matrix where identical markers detected in two compared samples was reported as one “hit”. The analysis (comparison) was performed with markers kept at a 0.1 decimal precision level. We ended up with 1000–1250 markers. The number of matches between two samples with the same marker was then calculated as numerical hits. Since the maximum possible obtainable number of hits varied somewhat due to the data screening method, the data (hits) are reported as % of maximum obtainable number of hits for each sample. A basic statistical examination of the distribution of the hit rates in our dataset revealed that there was a skewness (0.407) towards lower values. This will have the implication that mean hit rates calculated from few samples will have a tendency to be lower than when many samples are included. To test this we programmed a random generator to pick data from our complete dataset (n of hits = 628) in sets of 3 to 21 samples. The mean of these datasets varied between 200 and 455, and there was an increase up to 9–10 samples included. We therefore decided to apply only datasets with more than nine samples. This is also in accordance with proper statistical considerations, *i.e.*, to avoid low n values in order to gain more precise estimates of true population values [31]. We standardized the signal strengths according to Equation (3) and analyzed the data using principal component analysis (PCA). It is clear that this method does not precisely identify chemical components but only served as a measure of approximate similarities between samples.


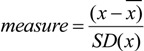
(3)

The method we applied here was also tested for reproducibility. One extract of *P. glacialis* was analysed 36 times the same day. By applying the method above we obtained a reproducibility of 89.6%, *i.e.*, *ca.* 90% of the markers were identical in all separate analyses at a 0.1 decimal precision level.

## 4. Conclusions

The aim of the present study was to reveal temperature and possibly size effects on the metabolic fingerprints of six common northern diatoms. A thorough test of the method applied was performed beforehand, and showed that the reproducibility of the method was *ca.* 90% at a one decimal precision level. In other words, the difference between replicates of the same sample with respect to *m/z* signals was *ca.* 10%. This means that a strict interpretation of our data, in this context, leads to the conclusion that the maximum similarities were up to 90% and that differences should be interpreted as having more than 10% variability.

Metabolomics literature comparing large amounts of metabolites across species cultivated at the same conditions is scarce. Nuclear magnetic resonance (NMR) analysis of metabolites has been shown to be able to differentiate between species of algae from different algal classes; a green algae and a dinoflagellate, but also between species from the same class as observed for a centric and a pennate diatom [32]. Also relevant in this context are studies with ultraperformance liquid chromatography (UPLC) coupled with time-of-flight mass spectrometry (TOF-MS) performed on the exometabolome of the diatom *Skeletonema marinoi* and *Thalassiosira pseudonana* [8] where the authors indicated that the exometabolite profile of the two diatoms *S. marinoi* and *T. pseudonana* differed extensively. Strains of the diatom *Chateoceros socialis*, originating from two geographically different areas, were also reported to be possible to differentiate between based on metabolite data [18,20].

A combination of metabolite and gene expression profiling studies of diatoms in different growth conditions and life cycle phases could be useful for an improved understanding of the highly diverse metabolism of diatoms.

In our opinion, our results demonstrate that chemical diversity increases at low temperatures (Figure 4). The main part of the available literature on algal chemistry and temperature deals with cell quotas and ratios of the main elements, *i.e.*, C, N and P, and the common conclusion seems to be that there is no clear effect of temperature [28,33,34]. However, Redalje and Laws have reported that N can decrease and C may increase with lowered temperatures [35]. This lack of consistency might be attributed to the fact that diatoms of the same species have variable chemistry and physiology [36]. The reason why diversity increases at lower temperature may lie in the fact that the species we tested can be found both in temperate water (Norwegian coast) and all the way up to the Arctic [21] and as such they have the ability to mobilize alternative metabolic pathways in order to adapt to the lowered temperatures. To get a better understanding of the underlying biochemistry of the changes in chemical profiles observed in our study, it would be very useful to identify what kind of metabolites that are up- and down-regulated.
